# Metabarcoding Reveals Fungal Diversity in the Root and Rhizosphere of *Isoëtes cangae*: An Endemic and Endangered Amazonian Species

**DOI:** 10.1155/ijm/1603429

**Published:** 2026-05-20

**Authors:** Aline Karl Araujo, Lupis Ribeiro Gomes Neto, Emiliano Nicolas Calderon, Rodrigo Lemes Martins, Allysson Buraslan Cavalcante, Francisco de Assis Esteves, Analy Machado de Oliveira Leite

**Affiliations:** ^1^ Institute of Biodiversity and Sustainability (NUPEM), Federal University of Rio de Janeiro, Macaé, Rio de Janeiro, Brazil, ufrj.br; ^2^ Vale S.A., Belém, Pará, Brazil

## Abstract

The roots of most plants worldwide are associated with diverse fungal communities present in the soil. This study provides a descriptive assessment of the fungal diversity associated with the roots and rhizosphere of *Isoëtes cangae*, an endangered species endemic to the Amazon. Two sampling campaigns were conducted during the rainy and dry seasons, with four adult individuals sampled per season along the southern margin of Amendoim Lake, Serra dos Carajás, Pará State, Brazil. Fungal community characterization was performed using an ITS amplicon metabarcoding approach on the Illumina HiSeq platform. A total of 31 fungal taxa were identified at the species level. The detected taxa included fungi commonly reported in the literature with different ecological lifestyles, such as saprotrophic and phytopathogenic groups. The most abundant phyla in both seasons were Ascomycota, Mucoromycota, and Glomeromycota. Alpha and principal coordinates analysis (PCoA) did not reveal statistically significant differences, indicating no significant variation in fungal diversity within or between seasons. Additionally, a high proportion of unclassified amplicon sequence variants (ASVs) was observed, highlighting gaps in reference databases and emphasizing the baseline nature of this study on fungal communities associated with the roots and rhizosphere of *I. cangae*.

## 1. Introduction

The roots of terrestrial plants host a wide spectrum of soil fungi that form diverse parasitic, mutualistic, and neutral associations [1, 2]. When in mutualistic associations, endophytes can contribute to the host plant, producing a multitude of substances that provide protection and, ultimately, survival value to the plant [3]. Freshwater aquatic plants, salt marshes, or mangroves follow this same trend; however, the microbiome of aquatic plants is not as explored [4].

Different plant species select specific microorganisms to live in their tissues [5]. This process is determined by an interaction between the genotype, phenotype, and geographic location of the host, which requires a deeper understanding of the assembly of specific microbial communities, with important considerations on the existence of endemisms [5].

According to van der Heijden et al. [6], further studies on fungal symbiotic associations in the closest extant relatives of land plants are still needed, as several evolutionary aspects remain unresolved. Arbuscular mycorrhizal symbiosis is considered ancestral among land plants and likely played an important role in their early evolution [7, 8].

Ferns and lycophytes are pioneer plants that may be useful for revegetation. *Isoëtes* Linnaeus is a cosmopolitan genus of heterosporous lycophytes; however, South America is emerging as a center of diversity, with Brazil being the richest region [9]. Recently, two new species of lycophytes, *Isoëtes cangae* (Pereira et al.) and *Isoëtes serracarajensis* (Pereira et al.), were discovered in the Brazilian part of the Amazon region. Both species are aquatic, but *I. serracarajensis* can survive in seasonal lakes, whereas *I. cangae* is found in a single permanent lake in the Amazonian region of Serra dos Carajás, Pará, Brazil [10].

Red Lists published in several European regions indicate that most *Isoëtes* species are highly threatened and require urgent protection. Climate periodicity changes, water quality degradation, solar radiation, temperature variation, and fluctuations in water level are among the main threats demanding conservation efforts [11, 12]. Despite the growing body of ecological knowledge surrounding *Isoëtes*, its natural distribution and interactions with soil fungi have received little attention [13]. To date, research has been limited to the bioprospecting of endophytic bacteria with plant growth‐promoting potential [14]. Here, we present the first descriptive survey of the rhizospheric and endospheric mycobiota associated with *I*. *cangae*, an endangered and endemic Amazonian species. As a secondary objective, seasonal sampling was conducted to capture temporal variation and maximize the detection of fungal diversity.

## 2. Methodology

### 2.1. Collection Location

Serra dos Carajás is a mountain complex located in the southeast of the state of Pará, characterized by its mineral wealth, rugged relief, and the presence of plateaus with isolated outcrops of ferruginous rocks [16]. The elevations of the Serra dos Carajás are known locally as Serra Norte and Serra Sul, two large sets where the main iron ore deposits are located within the Carajás National Forest [15]. This region has a rainy tropical with winter drought climate (AWi), according to Koppen′s classification, with an annual precipitation range of more than 2000 mm concentrated during a rainy season from November to March, whereas the dry season occurs between May and September [17]. The average temperature ranges from 25° to 26°C, but the absolute recorded values range from 15°C, from July to October, to 38°C in the remaining months of the year [18].

**Figure 1:**
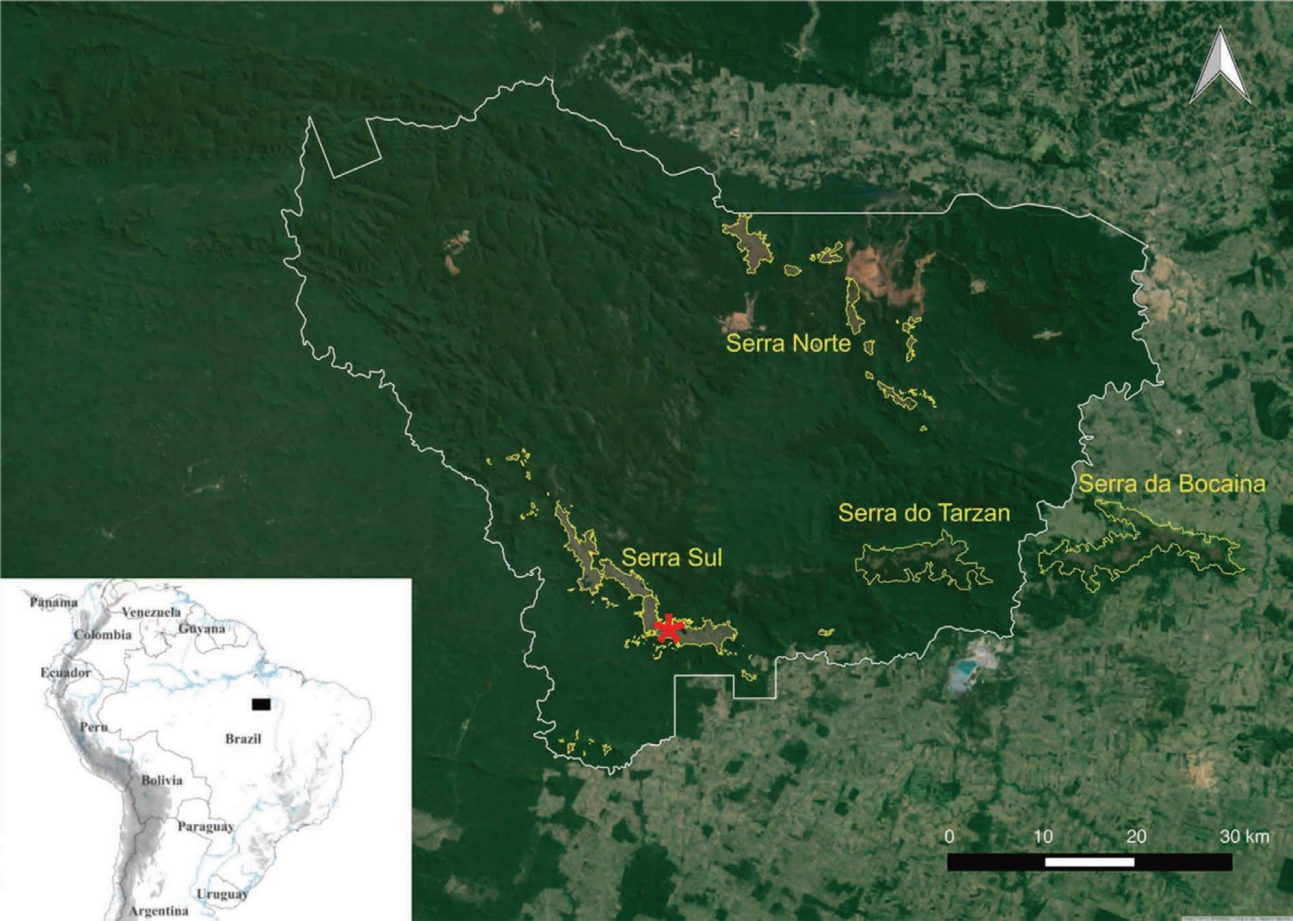
Serra dos Carajás geographic map comprising its mountain chains (Serra Norte, Serra Sul, Serra do Tarzan, and Serra da Bocaina) in Pará, northern Brazil. The white delimitation marks the limits of the Carajás National Park. The red star indicates the location of Amendoim Lake. *Source:* Adapted from ICMBio (2016) [15].

*I*. *cangae* is endemic to the permanent Amendoim Lake, located within the Carajás National Forest, a protected area in the southern Serra dos Carajás, in the southeastern Amazon region of Brazil (06°24 ^′^03.73 ^″^S; 050°22 ^′^23.27 ^″^W; 720 m a.s.l.) (Figure 1). Amendoim Lake is characterized as oligotrophic, with seasonal transitions to ultraoligotrophic conditions depending on annual hydrological variability [19]. The system exhibits high concentrations of iron (Fe) in both sediments and the water column, primarily due to its location atop extensive banded Fe formations [20]. Furthermore, the lake′s acidic pH is strongly influenced by the geochemical composition of the substrate [20], particularly the lateritic crust, which is enriched in Fe oxides.

In addition to *I. cangae*, the presence of another macrophyte, *Helanthium tenellum*, has also been recorded in the lagoon. According to Calderon et al. [21], *H. tenellum* naturally coexists with *I. cangae*, likely engaging in a competitive interaction for ecological niches and resources.

### 2.2. Collection of Plant Specimens

To account for seasonal variability and enhance the representativeness of the fungal community, two sampling campaigns were conducted during distinct climatic periods: the rainy season (March 2021) and the dry season (September 2021). Collection licenses (SISBIO Numbers 641873 and 871734) were issued by the Brazilian governmental environmental agency (ICMBio). Voucher specimens representing the sampled individuals were deposited in the Herbarium of the Federal University of Rio de Janeiro under the Accession Numbers RFA 41140, 41149, 41153, 41417, 41419, 41420, 41423, and 41424.

A total of eight adult specimens were collected, with four individuals sampled during each field campaign. Collections were conducted along the southern margin of the lagoon, where the highest concentration of individuals was observed at the time of sampling. Specimens were collected at the following geographic coordinates: 6.40162°S, 50.37280°W; 6.40152°S, 50.37294°W; 6.40132°S, 50.37309°W; 6.40072°S, 50.37320°W; 6.40111°S, 50.37314°W; 6.40055°S, 50.37322°W; 6.40058°S, 50.37310°W; and 6.40063°S, 50.37314°W.

Specimens without leaves were transported frozen to the Integrated Laboratory of Microbiology and Bioprocesses at NUPEM/UFRJ. The limited number of individuals collected during each sampling event reflects the species′ endemic nature and threatened conservation status. Sampling across multiple lagoon sectors was not feasible due to the difficulty of locating individuals.

### 2.3. Total DNA Extraction

Total microbial DNA was extracted after removal and disposal of the plant corms. Twenty‐five milligrams of the remaining material (roots plus adhering rhizospheric material) from each of the eight individuals were subjected to DNA extraction using the DNeasy PowerSoil kit (Qiagen), following the manufacturer′s instructions. This approach was chosen to recover total microbial DNA from mixed samples.

DNA concentration was quantified using a Qubit 2.0 fluorometer (Life Technologies, United States). ITS amplicons were sequenced using a paired‐end approach by Novogene Co., Ltd. (Sacramento, California, United States) on the Illumina HiSeq platform, employing the primer set ITS1‐1F (5 ^′^‐CTTGGTCATTTAGAGGAAGTAA‐3 ^′^) [22] and ITS2 (5 ^′^‐GCTGCGTTCTTCATCGATGC‐3 ^′^) [23].

### 2.4. Sequence Analysis and Statistics

Illumina HiSeq FASTQ files were imported into the QIIME2 platform (Version 2024.2) [24] and processed using the DADA2 algorithm [25]. All amplicon sequencing data were subjected to the same quality control pipeline, following an adapted version of the DADA2 ITS Pipeline Workflow 1.8 (https://benjjneb.github.io/dada2/ITS_workflow.html). Chimeric sequences were identified and removed. An amplicon sequence variant (ASV) table was generated and taxonomically assigned using the UNITE v9 database [26], which provides rDNA ITS‐based eukaryotic identification, including curated sequences from the International Nucleotide Sequence Databases Consortium (INSDC).

After removing low‐quality and short reads, a total of 672,781 ITS sequences were retained for downstream analyses, with an average of 84,097 sequences per sample. All sequencing data have been deposited in the National Center for Biotechnology Information (NCBI) under BioProject Accession Number PRJNA1132050.

Alpha and beta diversity analyses were performed using the MicrobiomeAnalyst (MA) platform [27]. Alpha diversity was estimated using Shannon and Observed richness indices and descriptively compared using an ANOVA test.

Beta diversity was assessed using Bray–Curtis dissimilarity and visualized by principal coordinates analysis (PCoA). Differences in community composition between seasons were explored using permutational multivariate analysis of variance (PERMANOVA), which is appropriate for multivariate ecological data and relatively robust to distributional assumptions under low replication [28].

Additional exploratory analyses of differential taxon abundance between seasons were performed using the nonparametric Wilcoxon rank‐sum test (*p* < 0.05).

## 3. Results and Discussion

After quality filtering, a total of 701,204 ASVs were retained, of which 672,781 were identified as nonchimeric and used for downstream analyses. For community structure analyses, the filtered ASV table was rarefied to an even sequencing depth of 72,945 reads per sample to reduce biases associated with unequal sequencing effort across samples. Rarefaction is widely used to standardize sequencing depth across samples. Although it may reduce statistical power due to subsampling and does not fully address the compositional nature of sequencing data, it remains a commonly applied normalization strategy for diversity‐based ecological comparisons [29].

### 3.1. Community Structure and Diversity

**Figure 2:**
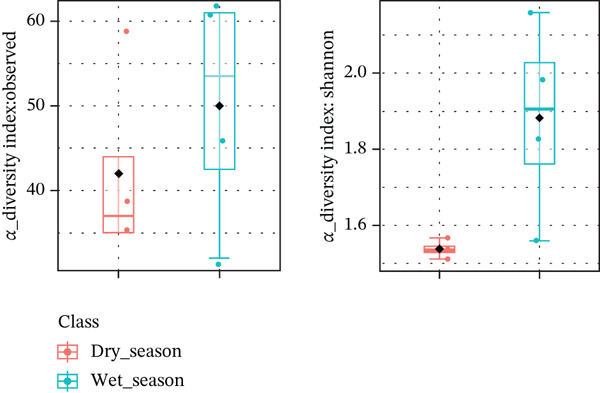
Alpha diversity of fungal communities associated with *Isoëtes cangae*, estimated using species richness (left) and Shannon index (right) for dry (*n* = 4) and rainy (*n* = 4) seasons. No statistically supported differences were detected between seasons (ANOVA test: species richness, *p* = 0.41; Shannon index, *p* = 0.07).

Alpha diversity was evaluated using species richness and the Shannon index (Figure 2). Both metrics showed numerically higher values in samples collected during the rainy season; however, these differences were not statistically significant (species richness: *p* = 0.41; Shannon index: *p* = 0.07). These results are therefore presented as descriptive observations rather than evidence of seasonal effects.

**Figure 3:**
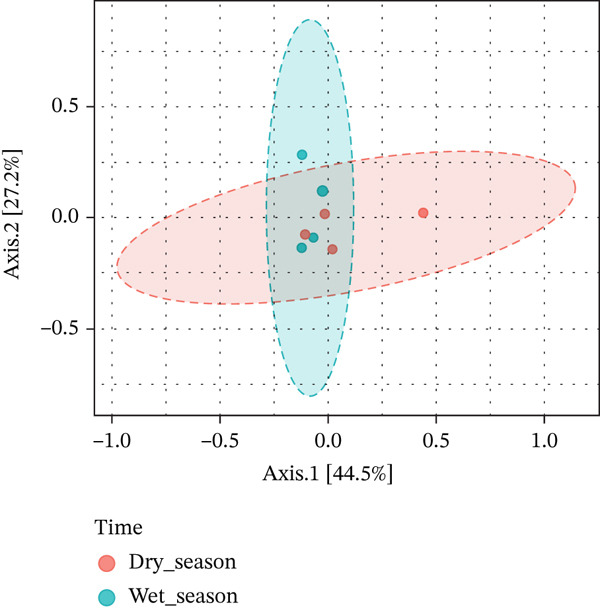
Principal coordinates analysis (PCoA) based on Bray–Curtis dissimilarity, illustrating the ordination of fungal communities associated with *Isoëtes cangae* across seasons. Samples represent dry season (*n* = 4) and rainy season (*n* = 4). No statistically supported separation between seasons was detected (PERMANOVA, *p* = 0.20).

Community structure was further assessed using beta diversity analysis based on Bray–Curtis dissimilarity. The PCoA ordination did not reveal a clear separation between samples from the rainy and dry seasons (Figure 3). Consistently, PERMANOVA did not detect statistically significant differences in community composition between seasons (*p* = 0.20). Within the scope of the present sampling design (*n* = 4 per group), no statistically supported seasonal differences in overall community structure were detected at the multivariate level. Therefore, the results should be interpreted as exploratory.

### 3.2. Taxonomic Composition of Fungal Communities

**Figure 4:**
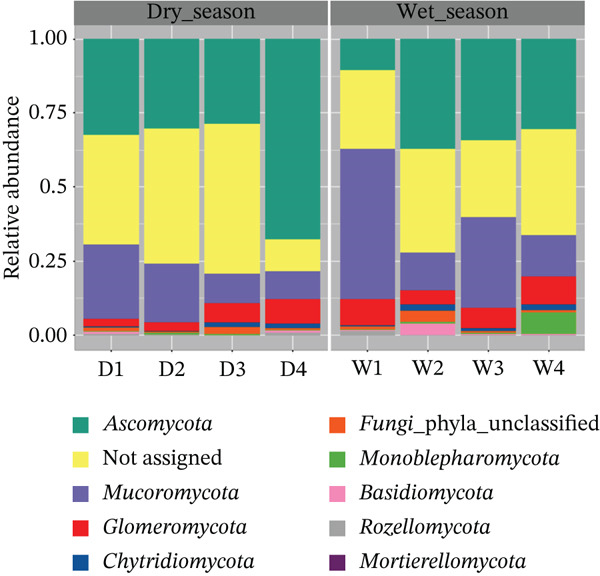
Relative abundance of fungal phyla across sampling periods, based on pooled root‐associated samples collected during the dry (*n* = 4) and rainy (*n* = 4) seasons.

Excluding representatives of an unidentified phylum, the three most abundant phyla in all samples were, respectively, Ascomycota, Mucoromycota, and Glomeromycota (Figure 4). It is noteworthy that 33.65% (196,385 sequences) of the total reads were classified as *Not_Assigned* at the phylum level. This proportion may be associated with limitations in current reference databases and taxonomic resolution, particularly for understudied fungal groups, and indicates that part of the detected diversity could not be confidently assigned. In addition, although widely used for fungal community profiling, this primer set is known to introduce taxonomic bias against certain fungal lineages, a limitation that should be considered when interpreting these results [30].

Ascomycota is recognized as the most diverse and species‐rich phylum in the kingdom Fungi, which comprises about 110,000 species [31]. The phylum Ascomycota includes yeasts and filamentous fungi, fungi that associate with algae and cyanobacteria to form lichen symbioses, mycorrhizal species, saprotrophs, and pathogens of plants and animals [32]. Therefore, its high relative abundance in the present dataset is consistent with previous reports [31].

The second most abundant phylum was Mucoromycota, which is a recently formalized phylum of fungi [33]. This phylum comprises three subphyla: Glomeromycotina, Mortierellomycotina, and Mucoromycotina [34]. Representatives of these subphyla are predominantly described as saprotrophs or as obligate plant symbionts [35–37].

The phylum Glomeromycota appeared as the third most abundant phylum. However, currently the name of the phylum Glomeromycota is not valid, since according to multigene phylogenetic analyses, this taxon is located as a member of the phylum Mucoromycota [34, 38]. Thus, the subphylum Glomeromycotina should be used to describe this taxon. Previous studies have reported that almost all fungi of the subphylum Glomeromycotina are obligate endomycorrhizal partners of plants, except for *Geosiphon pyriformis*, which forms a symbiotic association with endosymbiotic cyanobacteria [35, 39].

The phyla Chytridiomycota, commonly found in aquatic environments, and Basidiomycota, typically abundant in nature, were also identified (represented in blue and pink in the figure). The absence of these phyla among the most abundant groups may be associated with host‐related factors, although this relationship was not directly tested in the present study. According to Rimington et al. [40], fungal symbioses in lycophytes involve only members of the two Mucoromycota subphyla: Mucoromycotina and Glomeromycotina, with Glomeromycotina being the more prevalent. These observations highlight the need for further studies on fungal associations in lycophytes, since these were considered the best current analogues for the first vascular plants [41].

**Figure 5:**
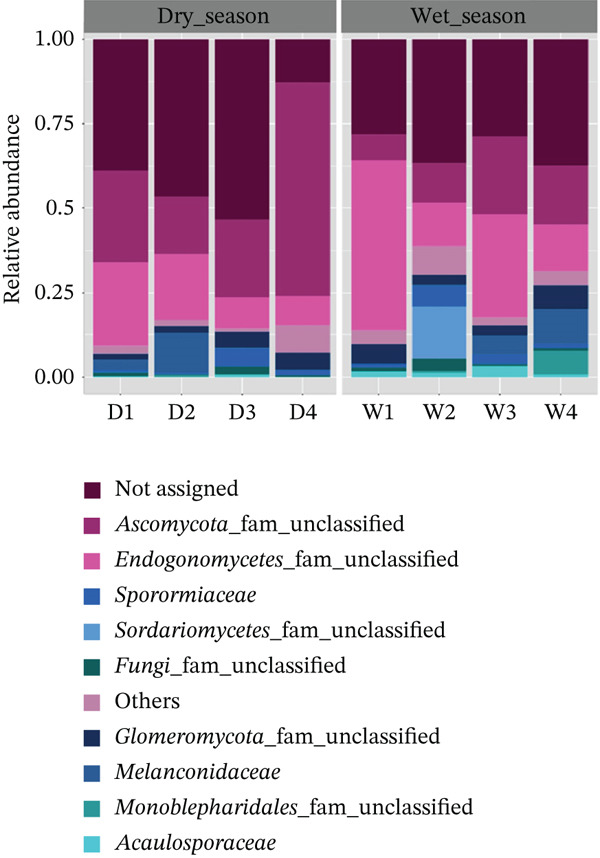
Relative abundance of fungal family across sampling periods, based on pooled root‐associated samples collected during the dry (*n* = 4) and rainy (*n* = 4) seasons.

Classification at the family level was not possible in some cases, reaching only the class or even phylum level (Figure 5). The difficulty in classifying some taxa may be related to the limited scope of available reference databases. As noted by Monteiro et al. [42], the fungal diversity associated with Amazonian canga ecosystems remains poorly characterized, with limited associated metadata. This highlighted the need for expanded taxonomic, ecological, and DNA barcoding efforts to more comprehensively reveal the true extent of fungal diversity in these environments. In addition to database limitations, the selection of the ITS marker may have introduced biases [30, 43]. Although the ITS region has been successfully used for soil fungal identification, primers targeting specific subregions (ITS1 and ITS2) have both advantages and limitations, and there is currently no consensus on the most suitable marker for each [30, 44].

A total of 31 fungal species were identified across the analyzed samples, and their previously described ecological roles, as reported in the literature, were summarized in Table 1. Most of these fungi have been characterized as phytopathogens or saprotrophs, and several species reported as opportunistic human pathogens were also detected.

**Table 1:** Fungal taxa detected in pooled root and rhizosphere samples of *Isoëtes cangae*. Ecological roles are putative and based on literature reports for related taxa.

Fungi identified at species level	Ecological function as reported in the literature
*Acremonium dichromosporum*	Mutualistic endophyte in plants [45]
*Candida parapsilosis*	Human pathogen [46]
	Ecological role [47, 48]
*Cladosporium sphaerospermum*	Saprobic [49]
*Cryptococcus uniguttulatus*	Environmental saprophyte [50]
*Fusarium solani*	Saprobic abundant in the soil [51]
	Food source for soil fauna [52]
	Facultative plant pathogen [53]
	Synergy with entomopathogenic nematodes [54]
*Malassezia restricta*	Although *M. restricta* is typically regarded as a human cutaneous commensal, its presence in soil, nematodes, and remote environments suggests that it may fulfill a saprophytic role or be adapted to dispersal via soil‐dwelling organisms [55–57]
*Meyerozyme* caribbean	Plant growth promoting endophyte [58]
	Biodegradation of pesticides [59]
	Plant biological control [60]
*Mortierella globalpina*	Saprobic and decomposer in the soil [61]
	Plant growth promoter [62]
	Biological control agent for nematodes [63]
*Nigrospora sphaerica*	Antimicrobial‐producing endophyte [64]
	Fungal antagonist [65]
	Environmental saprobe [66]
*Papiliotrema laurentii*	Environmental saprobe [67]
	Growth promotion agent [67]
	Biocontrol of phytopathogens [67]
	Degradation of natural/synthetic polymers [67]
*Phaeosphaeria oryzae*	Environmental saprobe [68]
*Rhizopus arrhythmia*	Global saprobity in soil and organic matter [69]
	High metabolic diversity [69]
*Rhodotorula mucilaginosa*	Environmental saprobe [70]
	Environmental remediation and pollutant tolerance [71]
	Promotion of plant growth in stressed soils [71]
*Saitozyme podzolic*	Soil saprobic [72]
	Edaphic indicator [72]
	Microbial carbon cycling [73]
*Schizophyllum commune*	Saprobic [74]
	Degradation of cellulose and hemicellulose [74]
	Microbial community modulation [75]
*Xenosonderhenioides indonesiana*	Saprobic [76]
*Zasmidium fructigenum*	Saprobic [77]
*Zygosporium oscheoides*	Saprobic [78]
*Aspergillus pseudogracilis*, *Cladosporium endophyticum*, *Conioscypha pleiomorpha*, *Dioszegia zsoltii*, *Leptospora macarangae*, *Leptospora phraeana*, *Microdochium* Colombian, *Neodevriesia agapanthus*, *Paraphaeosphaeria viridescens*, *Plectosphaerella slobbergiarum*, *Strelitziana eucalyptus*, *Strelitziana sarbhoyi*, *Symmetrospora sushi*	Poorly documented species within the genus

Some of the identified fungi have been previously reported as having beneficial roles in plant systems. For instance, *Mortierella globalpina* has been reported to promote tomato plant growth and to function as a biocontrol agent against root‐knot nematodes [63]. Similarly, *Papiliotrema laurentii* has been described in other systems as a biocontrol agent against phytopathogenic fungi and as a promoter of plant growth [67].

**Figure 6:**
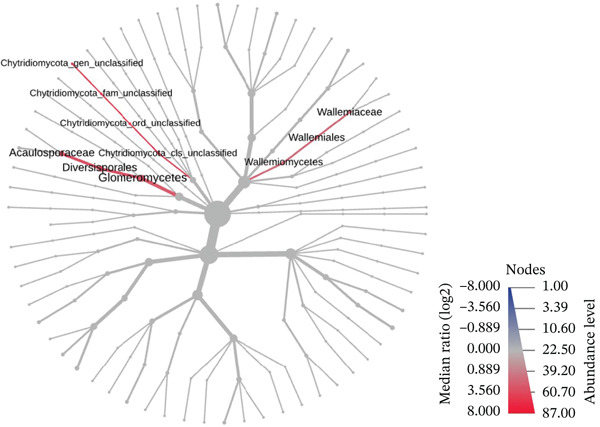
Exploratory differential abundance patterns between seasons based on the Wilcoxon rank‐sum test (*p* < 0.05, *n* = 4 per group). Colors indicate relative abundance trends, with red representing taxa showing higher relative abundance in the rainy season and blue representing taxa showing higher relative abundance in the dry season.

Although no statistically significant seasonal differences were detected in the multivariate ordination based on PCoA, taxon‐specific differences may occur without producing clear separation in community‐level ordination patterns, particularly under conditions of limited replication. As an additional exploratory step, a differential abundance analysis (Wilcoxon test, *p* < 0.05; *n* = 4 per group) was performed. The hierarchical taxonomic tree (Figure 6) suggested differences in the relative abundance of a limited number of taxa across seasons, including representatives affiliated with the phylum Chytridiomycota, as well as members of Glomeromycetes and Wallemiomycetes. Given the limited sample size, these results should be interpreted with caution and considered as preliminary indications of seasonal variation, particularly among less abundant taxa, and should primarily serve to guide future studies.

Chytridiomycota are frequently reported in aquatic and moist environments, where many species are described as saprobes or parasites [79, 80]. In the present dataset, a higher relative abundance of this group was observed during the wet season; however, no ecological inference can be drawn from this observation, as this relationship was not directly tested.

Wallemiomycetes (Basidiomycota), comprising the order Wallemiales, family Wallemiaceae, and the genus *Wallemia*, are generally described as xerophilic fungi adapted to conditions of low water activity [81]. In the present dataset, *Wallemia* spp. showed higher relative abundance during the rainy season in the root and rhizosphere compartments of *I*. *cangae*. This pattern may reflect context‐dependent ecological dynamics. However, given the exploratory nature of the analysis and the limited sample size, this observation should be interpreted with caution, and its ecological significance cannot be established within the scope of this study.

A relatively higher representation of Glomeromycetes was likewise observed during the rainy season. This class includes arbuscular mycorrhizal fungi (AMF), which have been reported in the literature as influencing plant nutrient acquisition and stress tolerance [82]. Seasonal variation in soil moisture and plant physiological activity has been associated with changes in AMF abundance in other systems [83, 84]. Nevertheless, because root tissues and rhizospheric soil were analyzed together and functional activity was not directly measured, the ecological relevance of this pattern in *I*. *cangae* remains uncertain and should be considered as a preliminary indication for future targeted investigation rather than evidence of a seasonal effect.

The predominance of saprotrophic, symbiotic, and stress‐tolerant fungal groups observed in this study may be associated with edaphic characteristics reported for Amazonian canga ecosystems, which are typically associated with ferruginous substrates, metal‐rich soils, low nutrient availability, and marked seasonal hydrological fluctuations [19]. In such environments, fungal communities potentially contribute to organic matter decomposition, nutrient mobilization, and plant stress mitigation. Although functional activity was not directly measured and root and rhizospheric compartments were analyzed jointly, these ecological interpretations provide a plausible framework for understanding the observed community patterns within this environmental context.

## 4. Conclusion

This study provides a first descriptive overview of the fungal communities associated with the roots and rhizosphere of *I*. *cangae*. Although taxonomic resolution may be limited by the current scope of fungal reference databases and potential primer‐related biases, 31 fungal taxa were identified at the species level, and community composition patterns were characterized at higher taxonomic ranks. The predominance of Mucoromycota (including Glomeromycotina) is consistent with previous reports of fungal associations in lycophytes, whose symbioses are mainly established with members of these groups.

Thus, this study provides a baseline dataset that expands current knowledge on fungal associations in an endangered and endemic Amazonian lycophyte species. These results provide a foundation for future studies focusing on functionally targeted groups, such as AMF, and for hypothesis‐driven investigations into the ecological relevance of fungal symbioses in *I. cangae*.

## Funding

This study was supported by the Vale S.A (4600068724).

## Conflicts of Interest

The authors declare no conflicts of interest.

## Data Availability

The data that support the findings of this study are available from the corresponding author upon reasonable request. The bioinformatic pipeline used in this study is available at https://github.com/lrg‐bio/Tutorials/blob/main/ITS_README.md. The raw reads of nucleotide sequence were deposited into the NCBI Sequence Read Archive (SRA) database (BioProject Accession: PRJNA1451240 and SRA Accessions: SRX32920447–SRX32920454).
